# Methodological Optimization of Supercritical Fluid Extraction of Valuable Bioactive Compounds from the Acidophilic Microalga *Coccomyxa onubensis*

**DOI:** 10.3390/antiox11071248

**Published:** 2022-06-25

**Authors:** Mari Carmen Ruiz-Domínguez, Elena Medina, Francisca Salinas, Waldo Bugueño, Juan-Luis Fuentes, Carlos Vílchez, Inés Garbayo, Pedro Cerezal-Mezquita

**Affiliations:** 1 Laboratorio de Microencapsulación de Compuestos Bioactivos (LAMICBA), Departamento de Ciencias de los Alimentos y Nutrición, Facultad de Ciencias de la Salud, Universidad de Antofagasta, Antofagasta 1240000, Chile; maria.ruiz@uantof.cl (M.C.R.-D.); elena.medina.perez@ua.cl (E.M.); francisca.salinas@uantof.cl (F.S.); waldo.bugueno@uantof.cl (W.B.); pedro.cerezal@uantof.cl (P.C.-M.); 2 Algal Biotechnology Group, CIDERTA and Faculty of Sciences, University of Huelva, 21007 Huelva, Spain; jlfuentes@dqcm.uhu.es (J.-L.F.); cvilchez@uhu.es (C.V.)

**Keywords:** *Coccomyxa onubensis*, supercritical fluid extraction, lutein, total phenols, antioxidant capacity, food applications

## Abstract

Microalgae grow in diverse environments and possess a great biotechnological potential as they contain useful bioactive compounds. These bioactive compounds can be obtained by selective and energy-efficient extraction methods. Various industries are using the supercritical fluid extraction (SFE) method to extract these valuable bioactive compounds. Hence, for the first time, we evaluated the effects of SFE on the recovery of bioactive and antioxidant compounds using *Coccomyxa onubensis*, a eukaryotic acidophilic microalga of potential relevance which can be used in the field of nutraceutical and functional foods. It was isolated from the Tinto River (Pyritic Belt, Huelva, Spain), a mining region in Spain. Variables such as extraction yield, lutein purity (LP) and recovery (LR), total phenols, and antioxidant capacity (Trolox equivalents antioxidant capacity method) were studied using a Box–Behnken design based on a response surface methodology along with the overall extraction curve fitted to a spline linear model. The effects of temperature (30, 50, and 70 °C), pressure (25, 40, and 55 MPa), and the percentage of co-solvent (0, 25%, and 50% *v*/*v* ethanol) on SFE were analyzed, resulting in the co-solvent and temperature as the most significant factors followed by the pressure. Under 70 °C, 40 MPa, and 50% *v*/*v* ethanol, *C. onubensis* reached a maximum of 66.98% of LR. The extracts were richest in total phenols and showed the maximum antioxidant activity (36.08 mg GAEs/g extracts and 2.237 mmol TE/g extracts, respectively) under similar pressure and co-solvent percentage values and different temperatures (30 and 70 °C, respectively). The extracts obtained in this study may have potential applications in the food, nutraceutical, and cosmetic industries. SFE is a highly efficient method to valorize microorganisms living in extreme environments, which are so far unexplored using green extraction methods.

## 1. Introduction

Microalgae are photosynthetic microorganisms that grow in diverse aquatic habitats such as lakes, ponds, rivers, oceans, and wastewater. They can tolerate a wide range of temperatures, salinities, pH values, and different light intensities and conditions in reservoirs or deserts, as well as can grow alone or in symbiosis with other organisms [1,2].

In recent years, microorganisms from various extreme environments have been analyzed for their biotechnological potential, and some of them have been used, especially in industrial processes, allowing their large-scale production to obtain different products [3,4]. Microalgae are a rich source of carbon compounds that can be used for the production of biofuels, health supplements, pharmaceuticals, and cosmetics [1,5]. They also have applications in wastewater treatment and atmospheric CO_2_ mitigation, as well as in the production of various valuable products, such as polysaccharides, carotenoids, phycobiliproteins, and polyunsaturated fatty acids (PUFAs), minerals, or vitamins [1,6,7,8].

*Coccomyxa onubensis* is a eukaryotic microalga isolated from the Tinto River in Huelva (Spain); the river has an acidic environment developed over centuries because of intensive mining activities aimed at pyrite extraction. The river has an average pH of 2.5 along 100 km and contains a high concentration of heavy metals such as iron, manganese, copper, and aluminum, as well as sulfate and nitrate [9,10,11]. This hostile, oxidative habitat forces *C. onubensis* to express adaptative antioxidant responses, including high carotenoid (mainly lutein) and PUFA synthesis [11,12]. Moreover, specific culture conditions have been found which explain the induction of lutein production or increased total lipid content and variations in the fatty acid profile of *C. onubensis*. The most prominent conditions include metal stress or mixotrophic media using urea [13,14], nutrient starvation media, or abiotic stress by salt or ultraviolet radiation [12,15].

Lutein has been described as an ocular protective agent, preventing macular degeneration via the reduction of reactive oxygen species formed by photochemical processes, and attenuating the blue light that strikes the retina in our eyes [16,17,18]. Navarro et al. [19] reported that *C. onubensis*, an acidophilic photosynthetic microalga, can be a nutraceutical source for functional foods. They proved that the microalga-powder-supplemented diets for laboratory rats exerted a considerable hypocholesterolemic and hypotriglyceridemic effect on the health of these laboratory rats, showing the potential of the antioxidant capacity of acidophilic microalgae in cardiovascular disease prevention. 

The correct choice of selective and energy-efficient extraction methods is an important stage in the industrial application of microalgal bioactive compounds. Traditionally, carotenoid compounds are recovered by conventional extraction techniques using organic solvents [20]. However, for health-related applications, severe restrictions on the use of these solvents have been proposed in addition to minimizing the environmental effect along with the cost-effective production of high-quality extracts [21,22]. An example of this is the use of non-conventional or green extraction technologies such as supercritical carbon dioxide extraction, also known as supercritical fluid extraction (SFE) using CO_2_. This technique is characterized by its high selectivity and effective bioactive compound recovery from multiple resources [20,22]. In this technique, CO_2_ is used as the main solvent, which is non-toxic, non-explosive, and easy to remove from extracted products. The polarity in SFE can be modified by the addition of a co-solvent or modifiers such as ethanol, directing extraction conditions to the maximum recovery of target compounds. Therefore, the food, cosmetics, and pharmaceutical industries are extensively applying SFE to obtain their respective products [22,23]. 

Hence, in this study, we focused on evaluating the effects of SFE on the recovery of bioactive compounds and their antioxidant activity in *C. onubensis* biomass: a distinctive acidophilic microorganism of high biotechnological potential. To our knowledge, we are the first to preliminarily evaluate the bioactive composition and antioxidant activity of acidophilic eukaryotic microalgae such as *C. onubensis* using SFE. This study shows the industrial applicability of extremophiles, minimizing the production cost by selecting species from hostile environments for the green and sustainable extraction of high added-value products.

## 2. Materials and Methods

### 2.1. Microalga Strain and Biomass Production

Microalgal biomass samples of *Coccomyxa onubensis* (SAG 2510, *C. onubensis*) were used in this study. The biomass was kindly donated by the research group Algal Biotechnology from the University of Huelva, Andalusia, Spain. *C. onubensis* is an acidophilic microalga that inhabits acidic mine drainages of the Pyritic Belt located in the north area of Huelva, in the southwest of Andalusia, Spain. *C. onubensis* was isolated and characterized by the Algal Biotechnology research group. The microalga was deposited in the Culture Collection of Algae at Goettingen University, in Germany [24]. The biomass samples sent from the University of Huelva were received in lyophilized form and packed in vacuum-sealed plastic bags and were stored at 15 ± 2 °C in darkness until further use. The biomass samples of *C. onubensis* were produced at the University of Huelva according to the following procedure. The culture medium was prepared in deionized water and contained the following mineral composition [11]: 3.95 g K_2_SO_4_, 0.1 g KCl, 0.5 g K_2_HPO_4_, 0.41 g MgCl_2_, 2.29 g KNO_3_, 0.01 g CaCl_2_, and 1-mL Hutner solution of trace elements, prepared as described by Garbayo et al. [11]. The pH of the medium was adjusted to 2.5 with sulfuric acid. The cultures were grown in a microalgae cultivation room at 25 °C. A mix of CO_2_ in air (5% *v*/*v*) was continuously bubbled into the cultures to supply carbon dioxide as an inorganic carbon source, thus promoting the photoautotrophic growth of the acidophilic microalga. The cultures were illuminated 24 h per day with 140 μmol photons m^−2^ s^−1^ of white light. Culture samples at the mid linear phase were harvested and the wet biomass pellets were lyophilized prior to delivery. The biomass was analyzed for metals and the following values were obtained (mg/kg dry weight): 0.0149, Mn; 0.1949, Fe; 0.0059, Co; 0.018, Ni; 0.5092, Cu; 0.3618, Zn. Other metals were present in concentrations below 0.001 mg/kg dry weight.

### 2.2. Chemicals

The main chemical compounds used in the SFE process were carbon dioxide (99% purity) and ethanol (99.5%). CO_2_ was purchased from Indura Group Air Products (Santiago, Chile) and ethanol was obtained from Merck (Darmstadt, Germany). Other chemicals used in this study were ultrapure water, lutein, gallic acid, 6-hydroxy-2,5,7,8-tetramethylchroman-2-carboxylic acid (Trolox, ≥97%), 2,2-azino-bis (3-ethylbenzothiazoline-6-sulfonic acid (ABTS ≥ 99%), and the Folin–Ciocalteu phenol reagent. The referred chemicals were all purchased from Sigma-Aldrich (Santiago, Chile). The latter company, Sigma-Aldrich, also supplied the chromatographic-grade organic solvents required for extraction: ethyl acetate, water, acetonitrile, and methanol. The solvents were heavy metals-free according to specifications.

### 2.3. Determination of the Particle Size of Powdered Coccomyxa onubensis Biomass

Samples of dry biomass of the acidophilic microalga were powdered using an analytical mill (A-11 basic Analytical, IKA Labortechnik, Staufen, Germany) for 20 s. Each grinding process was performed twice to ensure that the obtained results were reliable. The particle size distribution of powdered microalga was determined by using an electromagnetic and digital sieve shaker (CISA Cedaceria Industrial Sieving Technologies, BA200N model, Barcelona, Spain). Classifying the powdered biomass according to the particle size was possible thanks to the use of eight sieves made of stainless steel and equipped with a mesh of varying sizes: 10, 12, 16, 18, 35, 60, 120, and 230 according to ISO 3310–1 (ASTM E11 standard) equivalent to 1.80, 1.18, 1.00, 0.710, 0.500, 0.250, 0.125, and 0.063 mm of the mesh sizes. The procedure consisted of 15 min shaking the powdered biomass samples placed in the digital shaker, with an oscillation amplitude of 3 mm, after which the biomass got separated and retained in the sieves according to their particle sizes. Subsequently, the bottom was weighed and recorded. The mean particle diameter (Dp) of the *C. onubensis* powder was determined according to ASABE [25] using Equation (1). The mean value of the particle size of *C. onubensis* in the SFE process is shown and discussed in Section 3.1.

(1) $$D_{P}\;=\;exp\;\left\{\frac{\sum_{i\;=\;1}^{n}[\;\sqrt{W_{i\;\;}\cdot \log\;\left(d_{i}\cdot d_{i+1}\right)\;}]}{\overset{n}{\underset{i\;=\;1}{\sum }}W_{i}}\right\}$$

where *D_P_* is the mean particle diameter (mm); *d_i_* is the diameter of the sieve opening *i* (mm); *d_i_*_+1_ is the diameter of the sieve opening above sieve *i* (mm); *W_i_* is the retained mass (g); *n* is the total number of fractions. The so-obtained and classified samples were preserved in sealed plastic bags and stored in the refrigerator until use.

### 2.4. SFE 

A Speed Helix supercritical extractor (Applied Separation, Allentown, PA, USA) as described by Salinas et al. [26] was used in this work for the SFE experiments. For each extraction assay, 2.0 g of *C. onubensis* dry biomass were packaged into a 24 mL extraction vessel (nominal volume) occupying a volume of 2.54 mL (approximately 10.6% of the total volume of the extraction vessel) together with 1.0 mm glass beads. The temperature in each extracting process was maintained for 15 min by a heating jacket at a selected temperature for each run. The CO_2_ was pumped until the desired pressure was reached. This was maintained for 5 min (static time). The total extraction time was set at 60 min per the recommendations of a spline linear model described in Section 2.4 (Material and Methods), according to the yield and lutein recovery in the midpoint SFE condition. An electronic flow meter installed at the exit of a sample collection flask was used for the control of the CO_2_ flow rate. After the static time, the micrometric valve was adjusted to reach a CO_2_ flow rate of 2 L/min (~3.62 g/min). For the conditions that used a co-solvent, the pump was adjusted to 1.15 mL/min and 2.30 mL/min to reach a proportion of 25% and 50% over the CO_2_ flow rate, respectively. Finally, an N_2_-gas stream using Flexivap Work Station (Model 109A YH-1, Glas-Col, Terre Haute, IN, USA) was used for evaporating the residual ethanol present in the specific vials containing each SFE sample. Then, the dried extracts were stored at −20 °C and protected from light until further use.

### 2.5. Box–Behnken Experimental Design

The experimental conditions tested in this study (15 in total which are described in Section 3.3), were generated through a Box–Behnken design implemented in random run order. Three parameters, namely temperature, pressure, and ethanol concentration, were selected for the Box–Behnken design process. These parameters influenced directly on the response of target variables of the extraction process of *C. onubensis* biomass, including extraction yield (Y), lutein purity and recovery (LP and LR), total phenol content (TPC), and antioxidant capacity (Trolox equivalents antioxidant capacity (TEAC) method). The influence of each one of the above referred parameters on the SFE process was evaluated using the following values: 30, 50, and 70 °C for temperature; 25, 40, and 55 MPa for pressure; and 0, 25%, and 50% *v*/*v* for ethanol being used as a co-solvent. The experiments were performed in triplicates of *C. onubensis* (*n* = 3). The analyses of bioactive compounds extracted from the microalgal biomass and their antioxidant capacity are explained in the next sections.

### 2.6. Mathematical Modeling

Modeling the kinetic behavior of the extraction process has become a crucial step to designing an efficient SFE method. The so-called overall extraction curve (OEC) was obtained by plotting the extraction time versus the accumulated extract. The extraction kinetic was performed at the conditions given by the central point of the experimental design (50 °C, 40 MPa, 25% ethanol *v*/*v*). Each extract was collected at preselected time intervals of 5 to 15 min for a total of 15 points in the curve. The extraction yield (Y%) was calculated at each point of the curve. The experiment was performed in triplicate.

The OEC was fitted to a spline linear model. The spline model was defined from a base equation (Equation (2)), from which arose three specific equations that are described below this paragraph (Equations (3)–(5)). The OEC was fitted to the referred spline model. The spline modeling was performed by making use of the known procedures PROC REG and PROC NLIN of the free software SAS OnDemand for Academics. A Microsoft Excel-2016 spreadsheet was used to prepare figures from the fitted data obtained from Equation (5). Each one of the three extraction stages was represented by one of the fitted curves. The extraction stages differed in relation to the mass transfer mechanism that controlled them: (1) CER: stage determined by constant extraction rate; (2) FER: stage characterized by falling extraction rate, where the extraction process is essentially driven by convection and diffusion in the solid substratum; and (3) DC: stage essentially controlled by diffusion. These stages have been previously described in the literature [27]. The spline linear model allowed us to obtain the mass transfer rate for the CER period (M_CER_) and the time corresponding to the interception of two lines (t_CER_). The two stages left, FER and DC, were subsequently computed following the same approach. The experimental data obtained from the OEC were fitted into the spline model. The mass ratio of the solute in the supercritical phase at the equilibrium cell outlet (Y_CER_) was obtained by dividing M_CER_ by the mean solvent flow rate of the CER period, following the procedure performed by Ruiz-Domínguez et al. [28].

(2) $$m_{Ext}=\;\left(b_{o}-\overset{i=N}{\underset{i=1}{\sum }}C_{i}\;a_{i+1}\right)+\overset{i=N}{\underset{i=1}{\sum }}a_{i}t$$

For one straight line:

(3) $$m_{Ext}=b_{o}+a_{1}t\;\;\mathrm{for}\;t\;\leq \;t_{\mathrm{CER}}$$

For two straight lines:

(4) $$\;m_{Ext}={\;b}_{o}-t_{\mathrm{CER}}a_{2}+\left(a_{1}+a_{2}\right)t\;\;\mathrm{for}\;t_{\mathrm{CER}}\;<\;t\;\leq \;t_{\mathrm{FER}}$$

For three straight lines:

(5) $$m_{Ext}=b_{o}-t_{\mathrm{CER}}a_{2}-t_{\mathrm{FER}}a_{3}+\left(a_{1}+a_{2}+a_{3}\right)t\;\;\mathrm{for}\;t_{\mathrm{FER}}\;<\;t$$

where *m_Ext_* is the mass of the extract; *b*_0_ is the linear coefficient of the first line (CER); *a_i_* (*i* = 0, 1, 2, 3) are linear coefficients of lines; t is time (min); t_CER_ is CER time (min); and t_FER_ is FER time (min). *C_i_* for *i* = 1, 2 are the intercepts of these lines (C_1_ for the first and second lines, and C_2_ for the second and third lines).

Once the parameters were adjusted, these were used to calculate y_CER_ and y_FER_ from t_CER_ and t_FER_. Each recovery percentage value at a given time was calculated by Equation (6):

(6) $$Recovery\;\left(\%\right)=\frac{y_{t}}{y_{time\;final\;OEC}}\;\left(100\right)$$

### 2.7. Bioactive Compounds and Antioxidant Capacity

#### 2.7.1. Lutein Quantification 

Lutein extraction was performed using 0.05% (*w*/*v*) butylated hydroxytoluene (BHT) in methanol as an organic solvent. The optimal sample size for lutein extraction was 20 mg of dry biomass or 50 mg of supercritical fluid extracts. The obtained extracts were filtered (Ø = 0.22 µm filter), transferred into a chromatography vial, and immediately used for a lutein analysis (20 µL of injection volume). Lutein was quantified by liquid chromatography. An HPLC system (model 7100, Merck Hitachi LaChrom, Tokyo, Japan) equipped with a reverse-phase column (C18, 250 mm × 4.6 mm, 5 µm, Restek, Bellefonte, PA, USA) was used. The HPLC system was equipped with three pumps (flow rate of 1 mL/min) and a UV-Vis detector. The microalgal carotenoids, specifically lutein, were detected at 450 nm. Two solvents were used as a mobile phase: solvent A (ethyl acetate) and solvent B (acetonitrile-water 90:10). Each sample run took 17 min and proceeded according to the following solvent gradient: it started with 100% B for 10 min, followed by 50% B for 4 min, 40% B for 2 min, and ended with 100% B for 1 min. The free lutein concentration in the samples was calculated using the corresponding calibration curve obtained with pure lutein. The calibration curve was linear within the lutein concentration range 1–50 ppm (R^2^ = 0.9981); the lutein concentration being referred to dry biomass or weight of extract (LP). Lutein recovery (LR) was calculated by Equation (7):

(7) LR (%)=(Wc/Wt)·100 

where Wc is the mass of lutein (mg) extracted under any of the conditions described in this study, and Wt is the mass of lutein extracted conventionally (mg). The latter value was obtained using methanol (0.05% *w*/*v* BHT) as an extraction solvent. The biomass samples were added the referred solvent and placed in a shaker incubator at 300 rpm at 30 °C for 24 h. This allowed us to obtain an average lutein concentration of 3.14 ± 0.11 mg/g, expressed as mg of lutein per gram of dry weight of *C. onubensis* (benchmark extraction). The experiment was performed in triplicates (*n* = 3).

#### 2.7.2. Determination of TPC

The total phenolic content was analyzed by using the Folin–Ciocalteu method described by Ainsworth and Gillespie [29]. A volume of 20 µL of diluted extract (2.0 mg/mL) was mixed with 100 µL of 10% (*v*/*v*) Folin–Ciocalteu reagent and the mixture was kept at room temperature for 5 min. Subsequently, 75 µL of sodium carbonate solution (0.7 M) was added to the mixture which was then gently shaken for 1 min. The formation of a chemical complex between the phenolic compounds and the reagent yielded a blue color which was allowed to evolve for 60 min at room temperature (20 ± 2 °C). The procedure ended reading the absorbance at 765 nm on a microplate reader (BioTek Synergy HTX multi-mode reader, software Gen 5 2.0, Winooski, VT, USA). For calibration, gallic acid dilutions (0–2.0 mg/mL) were used as standards. Results were expressed as gallic acid equivalents (GAE)/g extracts. Each result was the average of three independent measurements.

#### 2.7.3. Determination of Antioxidant Capacity

The TEAC of the lutein-enriched extracts was quantified using the method described by Re et al. [30] with modifications proposed by Sánchez-Camargo et al. [31]. This method quantifies the chemical capacity of antioxidant substances present in the microalgal extracts to neutralize the free radicals produced by a radical-producing standard compound, 2,20-azino-bis (3-ethylbenzothiazoline-6-sulfonic acid) diammonium salt (ABTS^•+^). The radicals were produced by the chemical reaction between 7 mM ABTS and 2.45 mM potassium persulfate in the dark at room temperature for 16 h. The resulting ABTS^•+^ aqueous solution was diluted with 5 mM sodium phosphate buffer at pH 7.4 in order to reach an absorbance value of roughly 0.7 (± 0.02) at 734 nm. The antioxidant capacity of microalgal samples was measured in mixtures composed of 20 µL of sample and 180 µL of the ABTS^•+^ solution. The mixtures were placed in the wells of a 96-well microplate reader of a spectrophotometer. After 10 min of reaction, the absorbance decay at 734 nm was measured. Trolox was used as a reference standard. The TEAC was expressed as mmol Trolox equivalents (TE)/g extracts. Each result was the average of three independent measurements.

### 2.8. Statistical Analysis

Experimental designs and a data analysis were performed by response surface methodology (RSM) and the standardized Pareto chart (shown in Supplementary Material, Figures S1–S5) using the Statgraphics Centurion XVIII^®^ (Stat-Point Technologies, Inc., Warrenton, VA, USA) software. The effects of the factors on the response variables in the separation process were assessed using a pure error and considering a confidence interval of 95% for all the variables. The effect of each factor on the response variables and their statistical significance were analyzed by performing the analysis of variance (ANOVA). The results are presented in Supplementary Materials (Tables S1 and S2). The response surfaces of the respective mathematical coefficients were also obtained, and a *p*-value of ≤0.05 was considered significant. All analyses were performed in triplicates (*n* = 3). 

The mathematical relationship of the response with three factors, *X*_1_, *X*_2_, and *X*_3_, of the experimental design was approached by Equation (8):

(8) $$Z={\;\beta }_{0}+\beta_{1}X_{1}+\beta_{2}X_{2}+\beta_{3}X_{3}+\beta_{12}X_{1}X_{2}+\beta_{13}X_{1}X_{3}+\beta_{23}X_{2}X_{3}+\beta_{11}X_{1}^{2}+\beta_{22}X_{2}^{2}+\beta_{33}X_{3}^{2}$$

where *Z* = estimate response; β_0_ = constant; β_1_, β_2_, and β_3_ = linear coefficients; β_12_, β_13_, and β_23_ = interaction coefficients between the three factors; and β_11_, β_22_, and β_33_ = quadratic coefficients. A multiple regression analysis was performed to obtain coefficients and equations that can be used to predict responses.

## 3. Results

### 3.1. Particle Size

**Figure 1:**
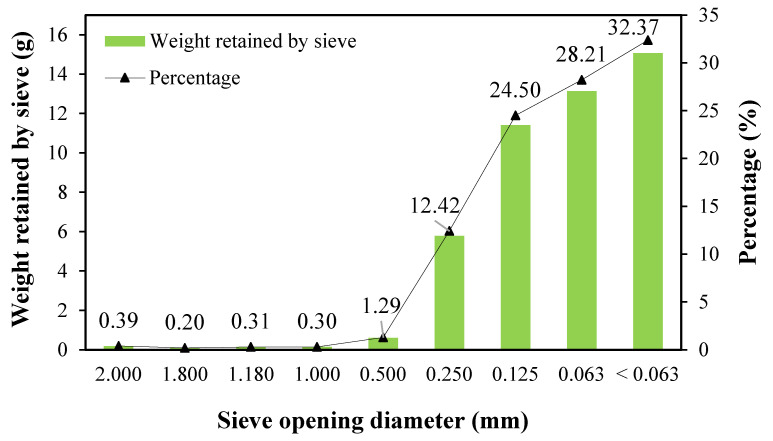
Particle diameter distribution.

Figure 1 shows the distribution of particles retained in each sieve. The figure shows that the retention process did not exceed 1.11% in the sieves with the largest opening, from 2.0 to 1.0 mm. A greater retention process started from the sieve with ϕ = 0.5 mm, which was 1.29%. The retention of the particles reached 97.5% from the sieve of ϕ = 0.250 mm to the blind bottom (ϕ < 0.063 mm). Therefore, a mean value of D_p_ = 0.079 ± 0.001 mm was expected for powdered *C. onubensis*. However, this means the value was low compared to those reported in other studies of the extraction of neutral lipids from microalgae using SFE, wherein the D_p_ ranged from 0.160 to 1.000 mm [32].

Partitioning, solubility, and diffusion inside the particle are the most important phenomena that govern the extraction rate in SFE [33]. The extraction bed is characterized by particle size and void fraction, which allows the calculation of the amount of CO_2_ between particles and its ratio to microalga feed [34]. Therefore, the extraction of bioactive lipids from the microalga *Nannochloropsis* sp. and the extraction of oil from the cyanobacterium *Spirulina* *(Arthrospira platensis)*, both freeze-dried and using supercritical fluids with CO_2_ at two temperatures, 40 and 55 °C, and pressures increased up to 70 MPa, were previously sieved to a maximum particle size of 0.35 mm and mixed with glass beads. The important result is that the mass transfer resistance increased with increasing pressure, which in turn increased with distance from the CO_2_ critical point [35,36].

The particle size should be as small as possible. However, a small particle size can restrict the diffusion of CO_2_ and create a channeling effect that reduces the efficiency in the extraction of the solutes of interest [37]. On the other hand, Crampon et al. [38] reported that with smaller particle sizes, the kinetics of extraction are more rapid, and the yields are higher. Therefore, the disintegration of cells is important in the recovery of intracellular products from algae.

### 3.2. Mathematical Modeling and Kinetic Curve of Coccomyxa onubensis SFE Extracts

The condition selected for kinetic study was the central point of the experimental design given by Box–Behnken. The conditions were as follows: 50 °C, 40 MPa, and CO_2_:ethanol (75:25 *v*/*v*) flow rates. These conditions were selected based on the yield results obtained for SFE from the acidophilic microalga *C. onubensis*.

**Figure 2:**
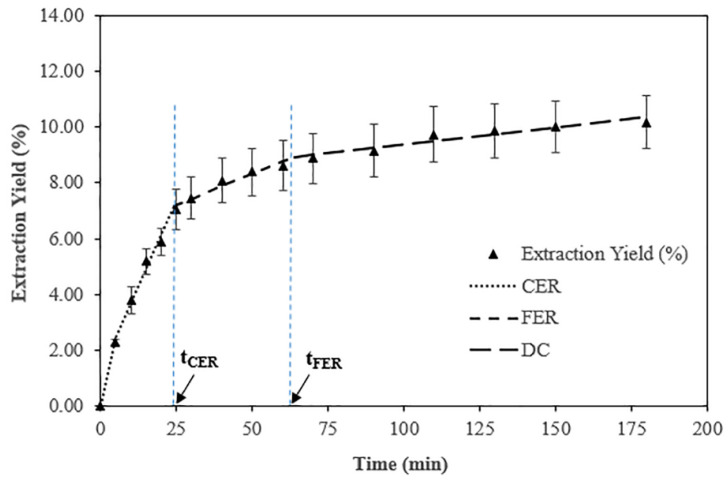
Overall extraction curves (OEC) of *Coccomyxa onubensis* by SFE at P = 40 MPa, T = 50 °C, CO_2_+ethanol (75:25 *v*/*v*) flow rate (3.1 g/min).

Figure 2 shows the overall extraction curves (OEC) of *Coccomyxa onubensis* dry biomass. A cumulative extract of 10.36% was obtained after 180 min of extraction. Dry biomass (~2.0 g) was used for extraction. The OEC plotted for dry biomass followed an SFE kinetic behavior similar to that reported by Jesus et al. [39] and Meireles [27]. The process began with the so-called CER period, in which the extraction of bioactive compounds that are easy to recover, both by the solvent and by the co-solvent, prevailed. The CER period was mainly controlled by the convection-type mass transfer in the vicinity of the fluid layer around the biomass particles. Once the CER period ended, a second transition period began which commonly takes place at a slower extraction rate being controlled by the typical mechanisms of mass transfer. This period has been referred to as the FER period. Once the solutes, whose accessibilities to extractant solvents were more favorable, became scarce within the microalgal biomass matrix, diffusion between the biomass particles became the main mass transfer mechanism during this last stage of the SFE, commonly called DC. In this way, the OEC behaved typically as a diffusion curvature with a minimum extraction rate.

Through the fitted data by spline linear model in Figure 2, the OEC parameters were estimated as shown in Table 1. The calculated t_CER_ was 24.33 min with the accumulated extracts of 7.18% and the recovery of 68.53%. This result is consistent with the results of previous studies that reported recovery values between 50% and 90% in the CER period [40]. The calculated t_FER_ was 63.86 min for the accumulated extract of 1.76% and a recovery of 17.32%, with a total accumulated extract of 8.94% up to this period and a total recovery of 85.85%. In the present study, a recovery above 85% was achieved in the FER period. The M_CER_ and M_FER_ values represented the extraction rate of the CER and FER periods, respectively [39], with values of 6.0 × 10^−3^ and 9.3 × 10^−4^ g/min. These values of the extraction rates were higher than the values reported for M_CER_ and M_FER_ in extracts of *Muriellopsis* sp. dry biomass using spray-drying (2.6 × 10^–3^ and 4.6 × 10^−4^ g/min) and freeze-drying (5.7 × 10^−3^ and 5.3 × 10^−4^ g/min) [41], respectively. When expressed in Y = mg extract/g biomass, the results were 70.27 and 17.56 for the CER and FER periods, respectively, which represented 1.91 × 10^−3^ and 3.0 × 10^−4^ g extract/g (CO_2_ 75% + ethanol 25%). This is the extraction ratio in the supercritical phase at the outlet of the bed, which had a greater extraction rate in the CER period.

In the final stage of the process or DC period (Table 1), lower values of all the parameters were observed for those values obtained in the previous CER and FER periods because the extraction already showed signs of exhaustion. Table 1 includes the coefficients of determination (R^2^) for each one of the extraction curve stages. The coefficients were determined using the spline linear model and their corresponding coefficients *b*_0_, *a*_1_, *a*_2_, and *a*_3_. A flow rate of 3.1 g/min was used for CO_2_ + ethanol (75:25% *v*/*v*), and a solvent to feed ratio (S/F) of 7.4 to 267.8 was selected.

As shown in Table 1, the intracellular lutein content of *C. onubensis* was fully recovered. This value is by far higher than those reported for lutein extraction from other species. The literature reports several examples of the latter. For instance, lutein recovery values of 47% were obtained from *Haematococcus pluvialis* dry biomass by means of using SFE-CO_2_ [42]. The extraction conditions were a CO_2_ flow rate = 3.62 g/min, temperature of 50 °C, and pressure of 40 MPa for a time of 120 min. The impact of the extraction conditions and the influence of the investigated microalgal species on the carotenoid recovery yield is evidenced by Yen et al. [43]. This study showed a higher lutein recovery value of 76.65% from *Scenedesmus* sp. dry biomass in a shorter time period of 60 min. The extraction conditions consisted of a much lower CO_2_ flow rate (1.45 g/min), 30% ethanol, a similar temperature of 47.5 °C, and the same pressure value of 40 MPa. 

This allows the highlighting of the relevance of the extraction optimization process as suboptimal extraction conditions address significant lutein losses which remain unextracted. 

According to our previous experience of carotenoid extraction optimization with other microalgae species as *Muriellopsis* sp. (MCH35), large CO_2_ to ethanol mixture ratios (85%/15%) were proven efficient for lutein [28]. The latter is consistent with similar studies for carotenoid extraction published by other authors for *Haematococcus pluvialis* [42], *Scenedesmus* sp. [43], and *Scenedesmus almeriensis* [44]. Interestingly, the maximum extraction rates were not systematically obtained within a fixed given time frame along the extraction period. Moreover, this may sometimes tend to happen by the end of the CER period, as reported by Meireles [27]. In addition, the costs of the extraction could in some cases be reduced if the process continues beyond the end of the CER period [45]. In our case, the extraction process was extended up to 60 min in order to ensure the maximum recovery of the extract, being greater than 85%.

### 3.3. The Yield of Coccomyxa onubensis SFE Extracts

The experimental extraction conditions and results of the Box–Behnken design from dry biomass of *C. onubensis* by the SFE process are shown in Table 2. 

As shown in Table 2, the factors were selected at three different experimental levels: temperature (30 °C, 50 °C, and 70 °C); pressure (25 MPa, 40 MPa, and 55 MPa); and the percentage of ethanol as the selected co-solvent (0%, 25%, and 50% *v*/*v*). The design matrix, evaluated for the response variable “extraction yield (%)” through the Statgraphic Centurion program (version XVIII), provided the general equation (Equation (9)) of the working model as follows:

**Yield** = −16.142 + 0.3252·T + 0.4188·P + 0.4389·EtOH − 0.0028·T^2^ + 0.0004·T·P − 0.0011·T·EtOH − 0.0048·P^2^ − 0.0047·P·EtOH + 0.0006·EtOH^2^ (9)

Based on the regression coefficients and *p*-value of the Box–Behnken design (Table S1 Supplementary Material) and the Pareto chart (Figure S1), the quadratic factor EtOH^2^ was not significant according to the adjustment made, as well as the interactions, namely T·P, T·EtOH, and P·EtOH ( underlined in the equation). Consequently, they were eliminated from the general equation (Equation (9)), which resulted in the following expression (Equation (10)):

**Yield** = −16.142 + 0.3252·T + 0.4188·P + 0.43890·EtOH − 0.0028·T^2^ − 0.0048·P^2^ − 0.0047·P·EtOH (10)

The best alternative for the chosen work surface was as follows: the temperature factor (T) was 50 °C, the pressure factor (P) was 55 MPa, and the ethanol (EtOH) co-solvent factor was 50% *v*/*v*. With this, an optimal evaluation was obtained for yield = 15.95%. According to ANOVA (Table S1 Supplementary Material), the value of R^2^ = 0.9936 and adjusted for the degrees of freedom was R^2^ = 0.9821.

This result is consistent with the maximum yield reached in run 13 with 16.32% *w*/*w*, followed by 13.28% and 13.27% *w*/*w* (runs 9 and 10, respectively). In all these experiences, the common extraction factor was the maximum extractant used, such as 50% *v*/*v* of ethanol. However, the temperature in these runs varied from 30 °C to 70 °C, and the pressure values ranged from low (25 MPa) to intermediate (40 MPa). The lowest yields which ranged from 0.79 to 2.99% *w*/*w* were significantly less and obtained without the presence of ethanol in the extraction process.

**Figure 3:**
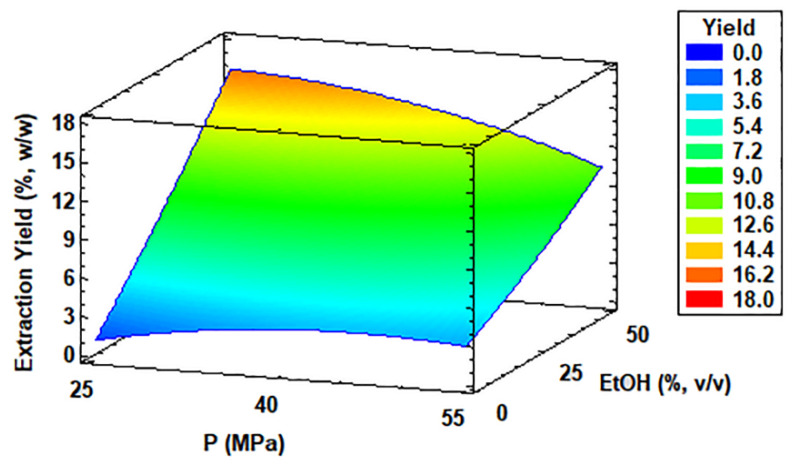
Response surface curve of the combined effects of temperature (30–70 °C), pressure (25–55 MPa), and ethanol as co-solvent (0–50% *v*/*v*) on extraction yield from *Coccomyxa onubensis*. Response surface curves were drawn from the viewpoint of the optimal temperature (50 °C). Abbreviation: temperature (T); pressure (P); and ethanol as co-solvent (EtOH).

Figure 3 also shows the results of extraction yields obtained from *C. onubensis* by response surface methodology (RSM). Particularly, the Pareto chart described in Supplementary Material (Figure S1) shows the significant factors and interactions for the extraction yield from the microalga in the SFE process. The ethanol co-solvent was the variable having a more positive effect on the extraction yield, followed by the interaction between solvent and pressure. As shown in Figure 3, RSM was drawn at an optimal temperature in the SFE process (50 °C), which improved the yield in *C. onubensis*. Supplementary Table S1 presents the statistical data and *p*-value in terms of the goodness of fit of the model for the extraction yield from *C. onubensis*.

Many studies confirmed the positive effects of the co-solvent, such as ethanol, on SFE yield and the solubility of a solute [46,47]. Pressure and co-solvent have been reported to compete with each other [46,48,49]. Our results showed this effect as a constant co-solvent percentage. The extraction yield decreased with an increase in pressure, and at constant pressure, the extraction yield improved as the extractant concentration increased. On the other hand, the density of the supercritical fluid can be modified by changing temperature and/or pressure. Because density is related to solubility, the solvent strength of the fluid can be modified to improve the extraction yield [50]. In the present study, the effect of temperature had a varied range and a non-significant effect on the extraction process (as shown in Figure S1 and Table S1). 

Mehariya et al. [44] optimized the main parameters that affected the SFE extraction from *Scenedesmus almeriensis,* which is a microalga described as a lutein producer. They used parameters similar to those of our studies, such as pressure (25–55 MPa), temperature (50 and 65 °C), a higher CO_2_ flow rate (7.24 and 14.48 g/min), and even a biomass pre-treatment for LP and LR. Particularly, the extraction yield was lower than our results, which reached its best extraction condition at 65 °C, 40 MPa, and a CO_2_ flow rate of 7.28 g/min with a value of 15.02 mg/g of yield (~1.5% *w*/*w*). Bueno et al. [51] reported the recovery of carotenoid from the microalga *Dunaliella salina* under compressed CO_2_ technologies. They used variables without a modifier (e.g., ethanol) such as pressure at 25 MPa, 32.5 MPa, and 40 MPa, and temperature of 15 °C, 30 °C, and 45 °C, wherein the best experimental condition for extraction yield was 45 °C, 25 MPa, and a CO_2_ flow rate of 12.70 g/min which was approximately two-folds less than our best result of yield. Furthermore, Reyes et al. [52] also worked with a Box–Behnken experimental design for optimization of the extraction yield, astaxanthin content, and antioxidant activity of *Haematococcus pluvialis* by SFE. They studied factors such as pressure (20–35 MPa), temperature (40–70 °C), and ethanol content in SFE (0–13%, *w*/*w*), similarly to our study. Their results showed improved extraction data compared to our results for *C. onubensis* at 55 °C, 20 MPa, and 13% ethanol with a value of 282.5 mg/g (28.25% *w*/*w* of yield). 

In general, our results are consistent with the results from previous studies, which confirmed that *C. onubensis* extracts showed a moderate extraction yield potentially rich in antioxidant compounds. This can be considered important in the novel food, nutraceutical, and cosmetic industries for the eventual use of acidophilic microorganisms.

### 3.4. LP and LR of Coccomyxa onubensis SFE Extracts

Table 2 also presents the purity and recovery values of lutein extracted from *C. onubensis* biomass using SFE. LP has been expressed in mg of this pigment (identified and quantified by HPLC) per gram of the extract. These data fluctuated from 1.49 to 25.39 mg/g of the extract under SFE conditions of 30 °C, 40 MPa, and 50% *v*/*v* ethanol and 70 °C, 55 MPa, and 25% ethanol, respectively. The optimal conditions described in the statistical software were 70 °C, 55 MPa, and 32.5% ethanol, with an optimal value of 25.28 mg/g. These results are consolidated with the statistical information added in the Supplementary Material (Table S1). The design matrix provided the following general equation (Equation (11)) of the working model:

**LP** = 24.959 − 0.147187·T − 0.7977·P + 0.2390·EtOH + 0.0009·T^2^ + 0.0025·T·P + 0.0055·T·EtOH + 0.0086·P^2^ + 0.0050·P·EtOH − 0.0138·EtOH^2^ (11)

Based on regression coefficients and the *p*-value obtained from the Box–Behnken design (Table S1 in Supplementary Material) and the Pareto chart (see Figure S2 in Supplementary Material), the EtOH factor, interaction T·P, and the quadratic factor T^2^ (they are underlined in the equation) were not significant according to the fit made. Consequently, they were eliminated from the general equation (Equation (11)), thus generating the following equation (Equation (12)):

**LP** = 24.959 − 0.147187·T − 0.7977·P + 0.0055·T·EtOH + 0.0086·P^2^ + 0.0050·P·EtOH − 0.0138·EtOH^2^ (12)

**Figure 4:**
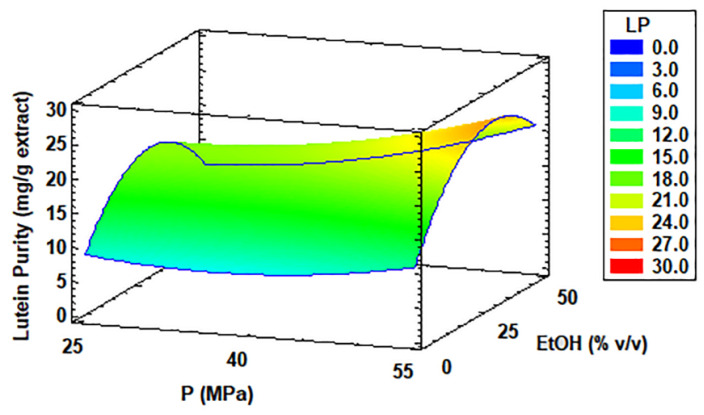
Response surface curve of the combined effects of temperature (30–70 °C), pressure (25–55 MPa), and ethanol as co-solvent (0–50% *v*/*v*) on lutein purity (LP) from *Coccomyxa onubensis*. Response surface curves were drawn from the viewpoint of the optimal temperature (70 °C). Abbreviation: temperature (T); pressure (P); and ethanol as co-solvent (EtOH).

The experiments without a co-solvent did not improve LP from *C. onubensis* (runs 6, 7, 11, and 12), with values ranging from 4.82 to 10.56 mg/g. This is complemented with the information given by Figure 4 (RSM). The presence of ethanol enhanced the solubility of the bioactive compounds in extractions [53,54]. Our results confirmed this up to a given maximum of extractant in the process. According to Table 2, the LP results under 25% *v*/*v* of ethanol (medium level) were better than those of 50% of the co-solvent (high level). An increase in temperature up to the highest level (70 °C) versus pressure up to the highest level (55 MPa) facilitated an increase in lutein purity in the microalga extracts. For instance, run 5 was approximately 1.4-fold higher for LP than run 4, where the temperature decreased to the lowest level (30 °C), with the same pressure and ethanol content (55 MPa and 25% co-solvent), respectively. This confirmed that an increase in temperature improves the diffusivity of CO_2_, thus resulting in enhanced extraction of bioactive compounds, as reported by Sapkale et al. [55].

The effects of SFE versus conventional methods on lutein extraction, LP, and LR are shown in Table 2. The concentration of lutein extracted by methanol maceration was 3.14 ± 0.11 mg/g, which was considered as mg of lutein per gram of dry weight of *C. onubensis*. This value was considered a benchmark for the extraction. Various microalgae species produce lutein, and their lutein content competes with that of marigold flowers; the genus of *Tagetes* sp. or *Calendula* sp. is one of the most used natural resources worldwide for xanthophyll extraction. The lutein content in marigold dry powder was in the range of 20–30 mg/g [56] as follows: *Chlorella fusca* (4.2–4.7 mg/g dry biomass); *Coelastrum proboscideum* (3.4–5.0 mg/g dry biomass); *Haematococcus pluvialis* (red phase, ~7.7 mg/g dry biomass); *Muriellopsis* sp. (MCH35) (3.45–4.20 mg/g dry biomass); or *Chlorella zofingiensis* (2.4–2.8 mg/g dry biomass) [28,57,58]. Thus, *C. onubensis* may be considered a novel biomass with a reasonable estimate for lutein contents, mostly in free lutein form, and can be improved according to the cultivation conditions.

The optimal conditions given in the statistical software for lutein extraction by supercritical fluids were 70 °C, 54.34 MPa, and 46.1% *v*/*v* ethanol, with an optimal value of LR = 66.39%. These results are consolidated with the statistical information added in the Supplementary Material (Table S1). The design matrix, evaluated for the response variables “LR (% *w*/*w*)”, provided the general equation of the working model (Equation (13)) given below:

**LR** = −23.3342 + 0.5381·T + 0.7420·P + 0.7138·EtOH − 0.0091·T^2^ + 0.00923·T·P + 0.0270·T·EtOH − 0.0129·P^2^ − 0.0001·P·EtOH − 0.0285·EtOH^2^ (13)

Based on regression coefficients and a *p*-value obtained from the Box–Behnken design (Table S1 in Supplementary Material) and the Pareto chart (Figure S3), we found that the *p*-value, the quadratic factors T^2^ and P^2^, and the interactions T·P and P·EtOH (they are underlined in the equation) were not significant according to the adjustment made. Hence, they have been removed from the general equation (Equation (13)), generating the following equation (Equation (14)):

**LR** = −23.3342 + 0.5381·T + 0.7138·EtOH + 0.0270·T·EtOH − 0.0285·EtOH^2^ (14)

**Figure 5:**
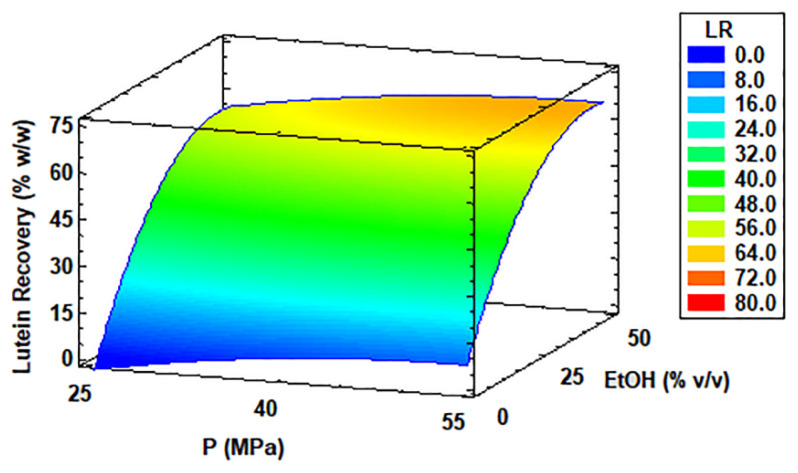
Response surface curve of the combined effects of temperature (30–70 °C), pressure (25–55 MPa), and ethanol as co-solvent (0–50% *v*/*v*) on lutein recovery (LR) from *Coccomyxa onubensis*. Response surface curves were drawn from the viewpoint of the optimal temperature (70 °C). Abbreviation: temperature (T), pressure (P), and ethanol as co-solvent (EtOH).

In Table 2, the maximum LR was 66.98% *w*/*w* which was obtained at 70 °C, 40 MPa, and 50% *v*/*v* ethanol. This was very similar to the optimal conditions obtained by the statistical software (optimal LR = 66.39% *w*/*w*). These results had the same trend as LP, which improved by increasing the ethanol concentration. However, the lowest results were obtained without extractant (e.g., LR in run 6 = 1.22% *w*/*w*). In general, temperatures above 50 °C increased LR under intermedia pressure (40 MPa). Figure 5 also shows the results of LR from the *C. onubensis* biomass extracted by SFE. Particularly, the Pareto chart in Figure S3 is shown in the Supplementary Material describing the following significant factors in the extraction process that ranged from higher to lower incidence: co-solvent, temperature, their interactions, and the quadratic factor of co-solvent. In most cases, all significant factors had a positive effect on the SFE process for LR. The pressure factor did not significantly affect the LR process by SFE, unlike the extraction yield results. However, the effect of the temperature factor was significant. On the other hand, Figure 5 shows the response surface curve from the point of view of the maximum temperature condition, which was 70 °C. Here, we observed that under a high temperature, the maximum value of extractant as ethanol had a significant effect in LR regardless of the pressure range. The Supplementary Material (Table S1) provides more statistical information to maximize LR from *C. onubensis* using SFE.

Compared with conventional extractions, supercritical fluid as green extraction has garnered increasing attention for the recovery of pharmaceutical or nutraceutical compounds because of their clean extracts, high recoveries, and the use of green solvents. This green extraction technology is being used in microalgal research. For instance, in a study performed by Molino et al. [58], CO_2_ supercritical fluid was used to perform astaxanthin and lutein extraction from *Haematococcus pluvialis* (red phase), with different variables such as the presence or absence of ethanol as a co-solvent. In addition, they studied other operational factors, such as extraction times (20, 40, 60, and 80 min); temperatures (50, 65, and 80 °C); and pressures (10, 40, and 55 MPa). Their biomolecules recoveries were higher than those obtained in our results (~92% *w*/*w* of astaxanthin and ~93% *w*/*w* of lutein), even though the pressure and temperature conditions used were very similar to those used in our study (65 °C, 55 MPa, and 12.5% *v*/*v* ethanol versus our optimal condition, 70 °C, 54 MPa, and 46% *v*/*v* ethanol). The only difference was the ethanol content because they used only one-third of the amount that was used in our experiments.

The polarity of free lutein is high, and the addition of polar co-solvents, such as methanol or ethanol, can increase the lutein extraction efficiency by SFE [18,53]. Our LR results were consistent with this. Therefore, they can be a promising lutein extraction method compared with organic solvent extraction.

### 3.5. TPC of Coccomyxa onubensis SFE Extracts

The optimal conditions described by statistical software for phenolic compound extraction were 70 °C, 54.9 MPa, 35.8% *v*/*v* ethanol, and an optimal value of 33.47 mg GAE/g extract. These results are consolidated with the statistical information and are shown in the Supplementary Material (Table S2). The design matrix, evaluated for the response variable “TPC (mg GAE/g extract)”, provided the following general equation of the working model (Equation (15)):

**TPC** = 4.8792 + 0.7968·T − 0.8526·P + 0.3422·EtOH − 0.0075·T^2^ + 0.0012·T·P + 0.0079·T·EtOH + 0.0093·P^2^ + 0.00813·P·EtOH − 0.0188·EtOH^2^ (15)

Based on the regression coefficients, the *p*-value of the Box–Behnken design (Table S2 in Supplementary Material), and the Pareto chart (Figure S4), we inferred that the factors P and EtOH, the quadratic factors T^2^ and P^2^, and the interactions T·EtOH and P·EtOH (both underlined in the equation) were not significant according to the fit made. Therefore, they were removed from the general equation (Equation (15)); thus, the following expression was obtained (Equation (16)):

**TPC** = 4.8792 + 0.7968·T − 0.0188·EtOH^2^ (16)

**Figure 6:**
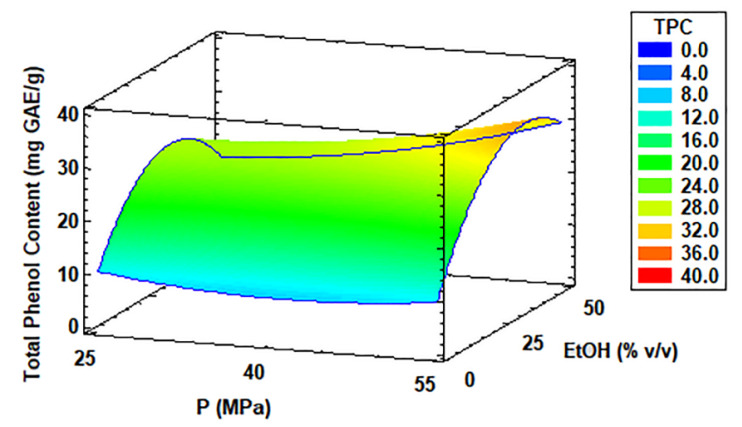
Response surface curve of the combined effects of temperature (30–70 °C), pressure (25–55 MPa), and ethanol as co-solvent (0–50% *v*/*v*) on total phenol content (TPC) from *Coccomyxa onubensis*. Response surface curves were drawn from the viewpoint of the optimal temperature (70 °C). Abbreviation: temperature (T), pressure (P), and ethanol as co-solvent (EtOH).

Table 2 also presents the TPC of the acidophilic microalga *Coccomyxa onubensis* by SFE, which ranged from 3.94 to 36.08 mg GAE/g extract, with run 5 yielding the greatest TPC at 70 °C, 55 MPa, and 25% *v*/*v* ethanol as extractant, and run 9 the least TPC at 30 °C, 40 MPa, and 50% ethanol. The optimal condition given by the software for maximizing TPC extraction was as follows: 70 °C, 55 MPa, 36% *v*/*v* co-solvent, and an optimal value of 33.47 mg GAE/g extract (a similar result to run 5). Figure 6 shows the response surface methodology of TPC based on the optimum temperature of 70 °C obtained by statistical software. It showed that with higher pressure values (from 40 to 55 MPa) and increased flow of the co-solvent, the TPC from *C. onubensis* was also higher. These results are presented in the Supplementary Material (Table S2) with information about the regression coefficients and *p*-value for the fit obtained by multiple linear regression.

Many studies have reported that the presence of ethanol favors the SFE-mediated extraction of the phenolic compounds from raw materials. Therefore, we considered that solubility increases with temperature. We also considered that polar compounds, such as proteins and carbohydrates, are better extracted using more polar solvents (e.g., water), whereas phenolic compounds and carotenoids are preferentially extracted using 100% ethanol [53]. For instance, Fabrowska et al. [59] studied the seaweed *Cladaphora glomerata* by SFE. The higher TPC content was obtained with 7.5% *v*/*v* ethanol in water as a co-solvent at 60 °C and 30 MPa, which reached a value of 16.10 mg GAE/g extract. Ruiz-Domínguez et al. [60] also reported the extraction of TPC by SFE from another microalga, *Isochrysis galbana*. A maximum TPC extraction yield of 157.16 ± 3.66 mg/g was achieved under the following conditions: 40 MPa, 50 °C, and 8% *v*/*v* ethanol. The extraction conditions selected in our study are quite similar to the latter ones. The presence of ethanol increased the extraction of phenolic compounds from the acidophilic microalga and was a significant factor in the SFE process (its quadratic form). Based on the results of the present study, the extraction patterns for *C. onubensis,* an extremophilic organism, showed similar trends to those reported by other chlorophytes. These results should be further consolidated with future studies.

### 3.6. Antioxidant Activity of Coccomyxa onubensis SFE Extracts

The optimal conditions given by statistical software were 70 °C, 55 MPa, 46.75% *v*/*v* ethanol, and an optimal value of 2.36 mmol TE/g extract. These results are consolidated with the statistical information presented in the Supplementary Material (Table S2). The design matrix, evaluated for the response variable “Antioxidant activity (mmol TE/g extract),” provided the following equation of the working model (Equation (17)):

**TEAC** = 1.9497 − 0.0094·T − 0.1059·P + 0.0092·EtOH + 0.0006·T^2^ − 0.0013·T·P + 0.0006·T·EtOH + 0.0022·P^2^ + 0.0007·P·EtOH − 0.0010·EtOH^2^ (17)

Based on the regression coefficients, the *p*-value of the Box–Behnken design (Table S2 in Supplementary Material); the Pareto chart (Figure S4); the quadratic factor T; and the interactions T·P, T·EtOH, and P·EtOH were not significant according to the adjustment made (they are underlined in the equation). Therefore, they were removed from the general equation (Equation (17)). Thus, the following expression was obtained (Equation (18)):

**TEAC** = 1.9497 − 0.0094·T − 0.1059·P + 0.0092·EtOH + 0.0022·P^2^ − 0.0010·EtOH^2^ (18)

**Figure 7:**
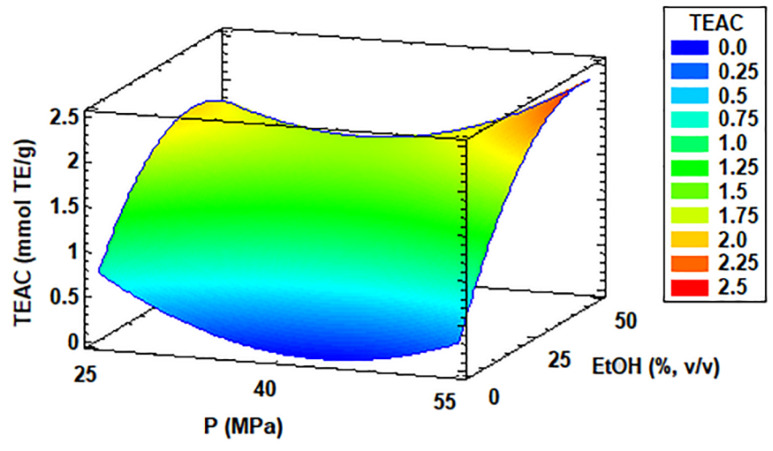
Response surface curve of the combined effects of temperature (30–70 °C), pressure (25–55 MPa), and ethanol as co-solvent (0–50% *v*/*v*) on antioxidant capacity (TEAC) from *Coccomyxa onubensis*. Response surface curves were drawn from the viewpoint of the optimal temperature (70 °C). Abbreviation: temperature (T), pressure (P), and ethanol as co-solvent (EtOH).

The antioxidant activity of the supercritical extracts obtained from *C. onubensis* cultures samples are summarized in Table 2. These results were obtained from the Box–Behnken experimental design for the optimization of the maximum antioxidant amount recovered. This variable was calculated by the TEAC method and expressed as micromoles of the Trolox equivalent (TE) to the extract weight (g). Normally, Trolox is used as a reference in the TEAC assay as a water-soluble vitamin E analog. Approximately, the antioxidant activity in the SFE extract of *C. onubensis* improved under high temperature, pressure, and percentage of co-solvent in the process. According to Table 2, the best TEAC value obtained experimentally was 2.237 mmol TE/g extract at 30 °C, 55 MPa, and 25% *v*/*v* ethanol. The optimal conditions obtained by software for maximizing TEAC in the supercritical process from the microalga were 70 °C, 55 MPa, and 46.7% *v*/*v* ethanol, with an optimum value equal to 2.36 mmol TE/g extract. These conditions were very similar to those obtained experimentally except for temperature. The Pareto diagram, again shown in Materials and Methods such as Figure S5, showed the maximum to minimum significant effects of the factors and interactions for the antioxidant activity in the microalga extracts. In Figure 7, the estimated response surface curve from the point of view of the optimum temperature used (70 °C) distinctly showed that the antioxidant activity improved under increased ethanol concentration (25 and 50%) and extreme pressure value sets (25 and 55 MPa).

Researchers have reported studies using TEAC assays to determine the antioxidant capacity of extracts. Fabrowska et al. [59] determined the valorization of *Cladophora glomerata, Ulva flexuosa,* and *Chara fragilis* as freshwater green macroalgae natural in Poland. They designed an experiment to optimize the extraction conditions by SFE for maximum response variables such as extraction yield, total carotenoids, total phenols, and antioxidant activity. Particularly, *C. glomerata* reached a value of 0.344 ± 0.012 mmol TE/g extract under SFE conditions of 40 °C, 30 MPa, and 7.5% of ethanol as a co-solvent. *U. flexuosa* showed the highest antioxidant activity (0.944 ± 0.137 mmol TE/g extract) in the studied seaweeds under the conditions of 40 °C, 30 MPa, and 11.4% ethanol. Our range of results (Table 2) was similar to their results. However, the best extraction condition of the antioxidant capacity in *C. onubensis* extracts was more than two-fold higher (run 4) than that of *U. flexuosa*. Goiris et al. [61] screened 32 microalgae samples to determine their antioxidant capacity using three antioxidant assays (including TEAC), total phenolic, and carotenoid content by conventional extraction methods. In this study, the extraction was performed in a one-step process using ethanol/water as the extractant solvent; alternatively, the microalgal biomass was extracted through a three-step procedure consisting of sequential extraction with hexane, ethyl acetate, and water. In general, *Phaeodactylum tricornutum*, *Botryococcus braunii*, *Neochloris oleoabundans*, *Chlorella* sp., and *Tetraselmis* sp. showed the highest antioxidant capacities using ethanol/waters as extractant ranging from 48.90 ± 1.30 to 69.40 ± 1.14 µmol TE/g extract. The use of green extraction techniques such as SFE lined up the extraction process with the circular economy principles. Thus, *C. onubensis* can be a potential novel source for natural antioxidants, and SFE proved to be a great extraction technique to improve the bioactive content and antioxidant activity present in acidophilic microalga *C. onubensis* extracts. 

## 4. Conclusions

To the best of our knowledge, this is the first study to characterize the antioxidant capacity in acidophilic microalgae, in particular *Coccomyxa onubensis*, focused on extracts obtained by SFE. We aimed to maximize the extraction process. In general, the co-solvent followed by the temperature were significant factors in the SFE process, which improved all extraction trials, especially when the maximum values of ethanol and temperature were used. The antioxidant activity of *C. onubensis* SFE extracts was high compared with other natural resources categorized as a potential source of antioxidants. To conclude, the supercritical extracts of the present acidophilic microalga showed a high antioxidant capacity based on the content of antioxidant compounds, which included carotenoids and phenolic compounds. An analysis of the metal content in the biomass must be performed to discard toxicity issues. In this respect, cultivation of the specific microalgal species must be performed under strict care to avoid the presence of toxic compounds in the extracts further obtained. This includes accurate quality control of the cultivation water and the nutrients’ source. The biomass produced and used in this study did not contain toxic levels of metals. Based on the above-mentioned characteristics, *C. onubensis* can be considered a novel, potentially valuable raw material for the cosmetic, food, nutraceutical, or pharmaceutical industries. Therefore, supercritical fluids-based green extraction processes can help increase the biotechnological value of extremophilic microorganisms. They can become an alternative, green extraction technique for the valorization of these unexplored microbial sources by isolating their bioactive components. 

## Figures and Tables

**Table 1:** Adjusted parameters of the spline linear model to supercritical fluid extraction (SFE) from *Coccomyxa onubensis* biomass at P = 40 MPa, T = 50 °C, and CO_2_ + ethanol (75:25 *v*/*v*) flow rate.

**Parameters**		**Stages of the General Extraction Curve**			
		**CER**		**FER**	**DC**
Time (min.)		24.33		63.86	180.0
Accumulated (%)		7.18		1.76	1.42
Accumulated Extract (%)		7.18		8.94	10.36
Recovery (%)		68.53		17.32	14.15
Total Recovery (%)		68.53		85.85	100.0
*M_EXT_* (g/min)		6.0 × 10^–3^		9.3 × 10^−4^	2.6 × 10^−4^
Y (mg extract/g biomass)		70.27		17.56	14.73
Y (g extract/g _(CO2 75% + ethanol 25%)_)		1.91 × 10^–3^		3.0 × 10^−4^	8.5 × 10^−5^
R^2^		0.9793		1.0000	1.0000
**Linear Coefficients of Spline model**	***b*_0_**		***a*_1_**	***a*_2_**	***a*_3_**
	1.2223		0.2449	–0.2003	–0.0324

$b_{0}$: Linear coefficient of the first line (CER); $a_{1},a_{2}\;\mathrm{and}\;a_{3}:$ Slopes of the lines 1, 2, and 3 corresponding to the periods CER, FER, and DC, respectively; $t_{\mathrm{CER}},\;\mathrm{and}\;t_{\mathrm{FER}}:$ Times in the intercepts of lines 1 and 2, and lines 2 and 3, respectively; $M_{EXT}\left(t\right):$ mass of the extract at time t; $Y_{t}$: Variable response for the consideration stage (CER, FER, and DC); $Y_{CGE}$: Variable response obtained from the sum of all the process times.

**Table 2:** Extraction yields (Y), lutein purity (LP), lutein recovery (LR), total phenol content (TPC), and antioxidant activity (Trolox equivalents antioxidant capacity (TEAC)) by supercritical fluid extraction from the acidophilic eukaryotic microalga *Coccomyxa onubensis* using the Box–Behnken experimental design. The general parameters are biomass loading = 2.0 g, CO_2_ flow rate = 3.62 g/min, and extraction time = 60 min.

Run	T (°C)	P (MPa)	EtOH (%, *v*/*v*)	Y (%, *w*/*w*)	LP (mg/g Extract)	LR (%, *w*/*w*)	TPC (mg GAE/g Extract)	TEAC (mmol TE/g Extract)
1	50	40	25	7.95	16.07	40.71	22.64	0.693
2	30	25	25	5.92	12.37	23.33	10.45	0.285
3	70	25	25	7.31	16.23	37.81	25.96	1.708
4	30	55	25	4.68	18.55	27.66	19.13	2.237
5	70	55	25	6.58	25.39	53.22	36.08	2.149
6	30	40	0	0.79	4.82	1.22	7.34	0.104
7	70	40	0	2.99	8.22	7.84	6.23	0.011
8	50	40	25	8.28	15.15	39.99	27.51	1.148
9	30	40	50	13.28	1.49	6.33	3.94	0.387
10	70	40	50	13.27	15.83	66.98	18.67	1.490
11	50	25	0	1.58	10.56	5.33	13.44	0.297
12	50	55	0	2.47	7.48	5.88	7.19	0.198
13	50	25	50	16.32	7.09	36.89	15.02	0.809
14	50	55	50	10.14	11.54	37.29	20.97	1.744
15	50	40	25	8.73	16.32	45.44	21.35	0.723

Abbreviations: temperature (T); pressure (P); ethanol (EtOH); gallic acid equivalent (GAE); Trolox equivalent (TE). Values presented are the mean of three determinations (±SD, *n* = 3).

## Data Availability

All of the data is contained within the article and the Supplementary Materials.

## Supplementary Materials

The following supporting information can be downloaded at: https://www.mdpi.com/article/10.3390/antiox11071248/s1, Figure S1: Pareto Chart of the combined effects of temperature (30–70 °C), pressure (25–55 MPa), and etha-nol as co-solvent (0–50% *v*/*v*) on extraction yield from Coccomyxa onubensis. Abbreviation: tem-perature (T); pressure (P) and ethanol as co-solvent (EtOH); Figure S2: Pareto Chart of the com-bined effects of temperature (30–70 °C), pressure (25–55 MPa), and ethanol as co-solvent (0–50% *v*/*v*) on lutein purity (LP) from Coccomyxa onubensis. Abbreviation: temperature (T), pressure (P), and ethanol as co-solvent (EtOH); Figure S3: Pareto Chart of the combined effects of tem-perature (30–70 °C), pressure (25–55 MPa), and ethanol as co-solvent (0–50% *v*/*v*) on lutein re-covery (LR) from Coccomyxa onubensis. Abbreviation: temperature (T), pressure (P), and ethanol as co-solvent (EtOH); Figure S4: Pareto Chart of the combined effects of temperature (30–70 °C), pressure (25–55 MPa), and ethanol as co-solvent (0–50% *v*/*v*) on total phenol content (TPC) from Coccomyxa onubensis. Abbreviation: temperature (T), pressure (P), and ethanol as co-solvent (EtOH). Figure S5: Pareto Chart of the combined effects of temperature (30–70 °C), pressure (25–55 MPa), and ethanol as co-solvent (0–50% *v*/*v*) on antioxidant capacity (TEAC) from Coccomyxa onubensis. Abbreviation: temperature (T), pressure (P), and ethanol as co-solvent (EtOH); Table S1: Regression coefficients and *p*-Value for Extraction yield, Lutein purity, and Lutein recovery in their original units and statistics for the fit obtained by multiple linear regression; Table S2: Regression coefficients and *p*-Value for Total phenol content and antioxidant activity in their original units and statistics for the fit obtained by multiple linear regression.
