# Supplementary material for: Methodological Optimization of Supercritical Fluid Extraction of Valuable Bioactive Compounds from the Acidophilic Microalga Coccomyxa onubensis
Source: Antioxidants (Basel). 2022 Jun 25;11(7):1248. doi: 10.3390/antiox11071248 (PMC9312109; doi:10.3390/antiox11071248)
Supplement: Supplementary file 1 [file antioxidants-11-01248-s001.zip › antioxidants-1704122-supplementary.pdf]

Methodological Optimization of Supercritical Fluid Extraction of Valuable Bioactive Compounds from the Acidophilic Microalga *Coccomyxa onubensis*

Mari Carmen Ruiz-Domínguez [1], **Elena Medina** [1], **Francisca Salinas** [1], **Waldo Bugueño** [1], **Juan-Luis Fuentes** [2], **Carlos Vílchez** [2], **Inés Garbayo** [2*], **Pedro Cerezal-Mezquita** [1]

[1] Laboratorio de Microencapsulación de Compuestos Bioactivos (LAMICBA), Departamento de Ciencias de los Alimentos y Nutrición, Facultad de Ciencias de la Salud, Universidad de Antofagasta, 1240000 Antofagasta, Chile

[2] Algal Biotechnology Group, CIDERTA and Faculty of Sciences, University of Huelva, 21007 Huelva, Spain

* Corresponding author: https://orcid.org/0000-0002-0656-4523

Supplementary material - Table S1. Regression coefficients and p-Value for Extraction yield, Lutein purity, and Lutein recovery in their original units and statistics for the fit obtained by multiple linear regression.

Terms of model	Extraction Yield (%, w/w)		Lutein purity (mg/g extract)		Lutein recovery (%, w/w)	
	**Estimate**	**p-Value**	**Estimate**	**p-Value**	**Estimate**	**p-Value**
Constant	-16.1420		24.9590		-23.3342	
A: T.	0.3252	0.0244*	-0.1472	0.0113*	0.5381	0.0010*
B: P	0.4188	0.0083*	-0.7977	0.0700	0.7420	0.2454
C: EtOH %	0.4390	0.0000*	0.2390	0.5327	0.7138	0.0005*
T × Temp.	-0.0028	0.0166*	0.0009	0.8011	-0.0091	0.2630
T × P	0.0004	0.6926	0.0025	0.5874	0.0092	0.3648
T × EtOH %	-0.0011	0.1291	0.0055	0.0867	0.0270	0.0046*
P × P	-0.0048	0.0193*	0.0086	0.2082	-0.0129	0.3632
P × EtOH %	-0.0047	0.0021*	0.0050	0.2030	-0.0001	0.9898
EtOH % × EtOH %	0.0006	0.2798	-0.0138	0.0013*	-0.0285	0.0016*
Statistic for the goodness of fit of the model						
R^2	0.9936		0.9364		0.9725	
adjusted R^2	0.9821		0.8219		0.9231	
RSD	0.3126		1.2589		5.5597	
P	0.7137		0.9424		0.9913	

R^2–determination coefficient. adjusted R^2. RSD–residual standard deviation. P-value of the lack-of-fit test for the model; * - significant coefficients of the model. T–Temperature. P–Pressure. EtOH %–Ethanol as Co-solvent

Supplementary material - Table S2. Regression coefficients and p-Value for Total phenol content and antioxidant activity in their original units and statistics for the fit obtained by multiple linear regression.

**Terms of model**	**Total Phenol Content (mg GAE / g extract)**		**Antioxidant activity (TEAC) (mmol TE / g extract)**	
	**Estimate**	**p-Value**	**Estimate**	**p-Value**
Constant	4.8792		1.9497	
A: T.	0.7968	0.0196*	-0.0094	0.0406*
B: P	-0.8526	0.2327	-0.1059	0.0129*
C: EtOH %	0.3422	0.1334	0.0092	0.0066*
T × T	-0.0075	0.2824	0.0006	0.1906
T × P	0.0012	0.8871	-0.0013	0.0545
T × EtOH %	0.0079	0.1611	0.0006	0.1048
P × P	0.0093	0.4424	0.0022	0.0242*
P × EtOH %	0.0081	0.2613	0.0007	0.1478
EtOH % × EtOH %	-0.0188	0.0054*	-0.0010	0.0129*
Statistic for the goodness of fit of the model				
R^2	0.9002		0.9437	
adjusted R^2	0.7208		0.8423	
RSD	2.3916		0.5337	
P	0.7128		0.6075	

R^2–determination coefficient. adjusted R^2. RSD–residual standard deviation. P-value of the lack-of-fit test for the model; * - significant coefficients of the model. T–Temperature. P–Pressure. EtOH %–Ethanol as Co-solvent

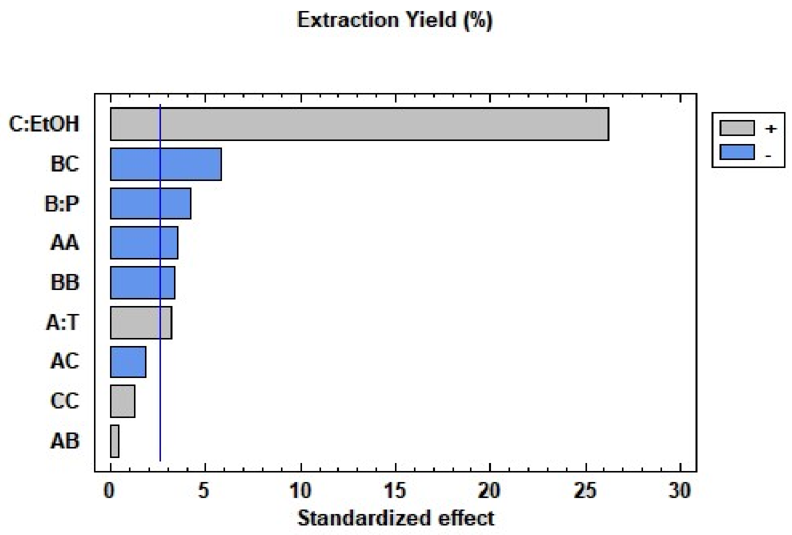

Figure S1. Pareto Chart of the combined effects of temperature (30–70 °C), pressure (25–55 MPa), and ethanol as co-solvent (0–50% v/v) on extraction yield from *Coccomyxa onubensis.* Abbreviation: temperature (T); pressure (P) and ethanol as co-solvent (EtOH).

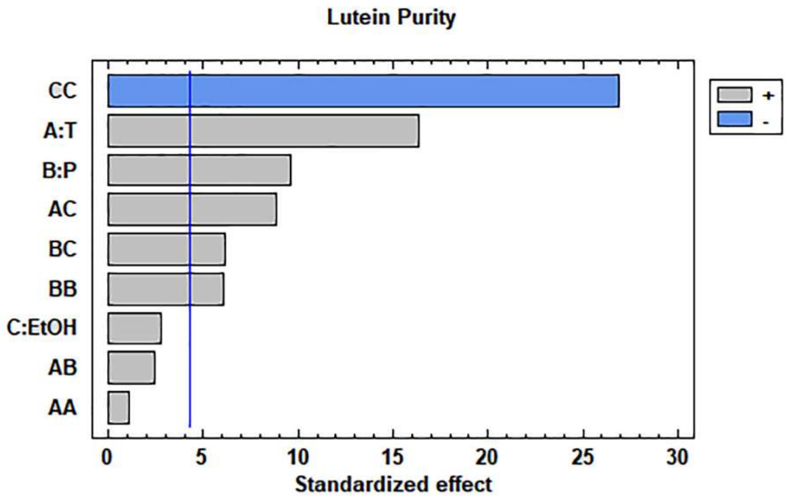

Figure S2. Pareto Chart of the combined effects of temperature (30–70 °C), pressure (25–55 MPa), and ethanol as co-solvent (0%–50% v/v) on lutein purity (LP) from *Coccomyxa onubensis.* Abbreviation: temperature (T), pressure (P), and ethanol as co-solvent (EtOH).

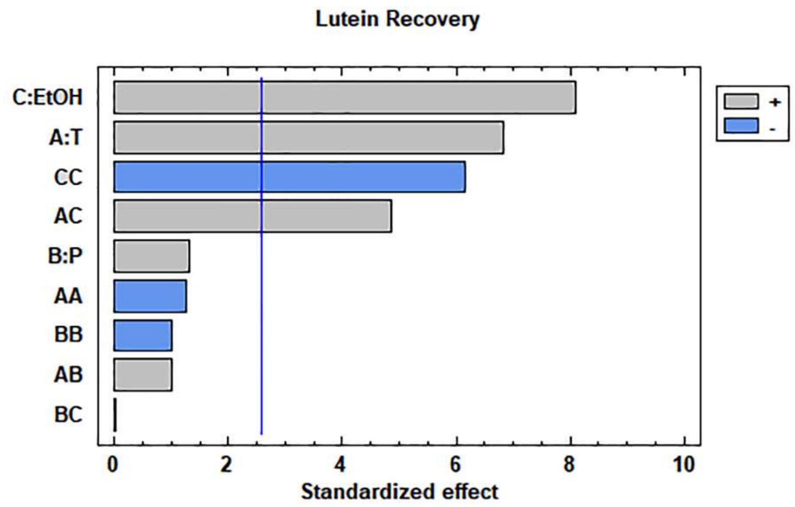

Figure S3. Pareto Chart of the combined effects of temperature (30–70 °C), pressure (25–55 MPa), and ethanol as co-solvent (0–50% v/v) on lutein recovery (LR) from *Coccomyxa onubensis*. Abbreviation: temperature (T), pressure (P), and ethanol as co-solvent (EtOH).

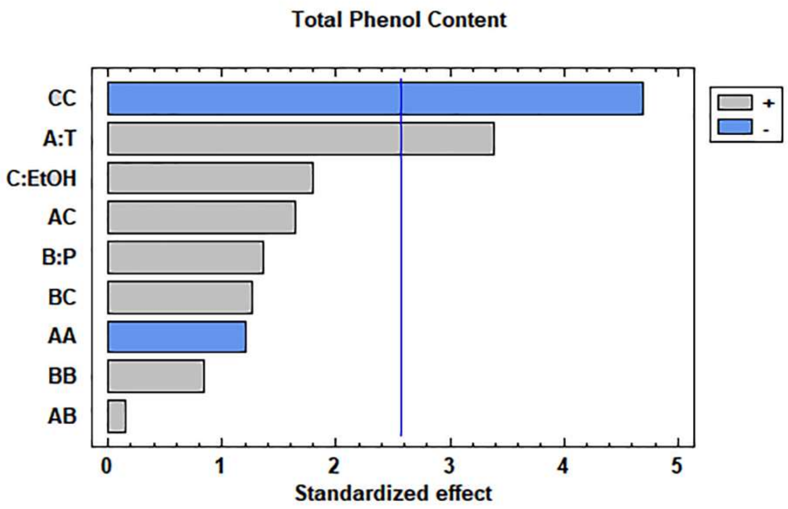

Figure S4. Pareto Chart of the combined effects of temperature (30–70 °C), pressure (25–55 MPa), and ethanol as co-solvent (0–50% v/v) on total phenol content (TPC) from *Coccomyxa onubensis*. Abbreviation: temperature (T), pressure (P), and ethanol as co-solvent (EtOH).

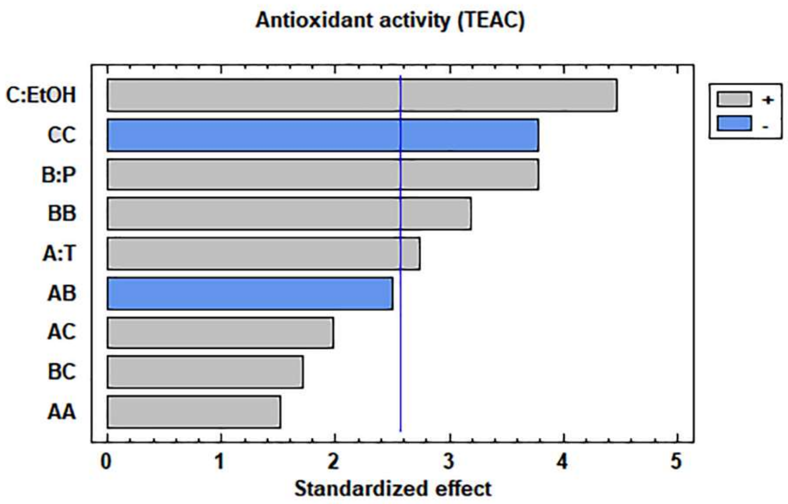

Figure S5. Pareto Chart of the combined effects of temperature (30–70 °C), pressure (25–55 MPa), and ethanol as co-solvent (0–50% v/v) on antioxidant capacity (TEAC) from *Coccomyxa onubensis*. Abbreviation: temperature (T), pressure (P), and ethanol as co-solvent (EtOH).
